# Periodization of altitude training: A collective case study of high-level swimmers

**DOI:** 10.3389/fphys.2023.1140077

**Published:** 2023-02-20

**Authors:** J. M. González-Ravé, J. A. Castillo, F. González-Mohino, D. B. Pyne

**Affiliations:** ^1^ Sports Training Laboratory, Department of Physical Activity and Sport Sciences, University of Castilla la Mancha, Toledo, Spain; ^2^ National Institute of Physical Education in Barcelona, Barcelona, Spain; ^3^ Facultad de Ciencias de la Vida y de la Naturaleza, Universidad Nebrija, Madrid, Spain; ^4^ Research Institute for Sport and Exercise, Faculty of Health, University of Canberra, Canberra, ACT, Australia

**Keywords:** elite, swimming, integrated periodization, altitude, season

## Abstract

The objective was to quantify parallel changes in performance and physiological measures in relation to periodization of sequential altitude training over a season in elite swimmers. The altitude training of four female and two male international swimmers in selected seasons was examined using a collective case study approach. All swimmers were a medalist in World (WC) and/or European Championships (EC) 2013, 2014, 2016 and 2018 in short or long course competition. A traditional periodization model was employed using three macrocycles with 3–4 altitude camps (duration 21–24 days each) scheduled over the season, following a polarized training intensity distribution (TID) with a volume ranged between 729 km and 862 km. The timing of return from altitude prior to competition was between 20–32 days, with 28 days the most common period. Competition performance was assessed with major (international) and minor (regional or national) competitions. Hemoglobin concentration, hematocrit, anthropometric characteristics, were measured before and after each camp. Competition performance following the altitude training camps improved by 0.6% ± 0.8% (personal best time; mean ± SD) (95% confidence limits (CL) 0.1%–1.1%), 1.6% ± 0.7% (95% CL 1.2% to 2.0%) (season best time) and 1.6% ± 0.5% (95% CL 1.3%–1.9%) (previous season time). Hemoglobin concentration increased 4.9% from pre-to post-altitude training camps, while hematocrit increased by 4.5%. The sum of six skinfolds reduced by 14.4% (95% CL 18.8%–9.9%) and 4.2% (95% CL 2.4%–9.2%) for the two males (EC), and by 15.8% (95% CL 19.5%–12.0%) for two females (WC). Three to four altitude training camps in a competitive season, around 21–24 days of duration, scheduling the last return between 20–32 days before the main competition, integrated in a traditional periodized sequence, can induce worthwhile improvements in international swimming performance, hematological parameters, and anthropometric characteristics.

## 1 Introduction

Altitude training is widely used by elite coaches and athletes to enhance competition performance. In the live high-train high (LHTH) altitude training model, athletes typically live and train for 2 to 4 weeks at altitude to prepare for a major competition (Saunders et al., 2019). This type of training can be employed to promote hematological adaptations, and training and competitive performance for an elite swimmer (Mujika et al., 2019; González-Ravé et al., 2022). Studies show that multiple exposures to moderate altitude (1800–2,500 m) can increase hemoglobin (Hb) mass in swimmers (Wachsmuth et al., 2013; González-Ravé et al., 2022). However, there are conflicting views on the actual physiological and performance effects of altitude training, and the underlying hypoxic and training dose required including the degree of hypoxia, duration of exposure to hypoxic conditions, exercise intensity and inter-individual variability (Mazzeo, 2008). Hypoxia is defined as the reduction of oxygen (O_2_) content or pressure at the cellular level. It has two subtypes: hypobaric hypoxia, characterized by an atmospheric pressure lower than 760 mm Hg and a fraction of inspired O_2_ (FiO_2_) of 20.9%; and normobaric hypoxia, with a barometric pressure of 760 mm Hg and a FiO_2_ of less than 20.9% (Fernández-Lázaro et al., 2020).

Studies of altitude training of competitive swimmers are difficult to organise in a traditional randomized controlled trial design. Consequently, altitude studies have predominantly been observational in nature, limited to physiological, hematological or immunological measures only, involved well-trained (Watanabe et al., 2019) but not elite swimmers, employed simulated (hypoxia training) (Camacho-Cardenosa et al., 2020), or restricted to discrete aspects of performance, time-trial performance (Rodríguez et al., 2015), or a single training/exposure intervention of 2–4 weeks in duration. Only a few studies have examined altitude in the context of international competition performance. Studies that have measured hematological and competition performance in parallel show conflicting results, variously reporting no direct transfer (Gough et al., 2012), or a small benefit (Wachsmuth et al., 2013), of increased hemogloblin concentration with performance. While these studies are informative, swimming coaches, sports science practitioners and researchers are seeking more specific details in the top cohort of swimmers. How results and the effects of various training interventions employed with lower-level athletes translate to their international counterparts is not always clear. Finally, little attention has been paid to the periodization of training in these studies despite the central importance of programming and periodization in high-level swimming programs (González-Ravé et al., 2021).

Two primary forms of periodization have been described in the sports science literature: traditional and block. Traditional periodization can also adopt a reverse form of distribution in intensity. Block periodization has two subtypes, single goal or factor (individual sports) and multiple goals or factors (team sports) (Stone et al., 2021). The most common periodization used in swimming is traditional periodization, (Hellard et al., 2019; González-Ravé et al., 2021), typically involving 1 to 2 macrocycles divided into 4 to 6 mesocycles allowing the programming of 3–4 altitude training camps through the season (González-Ravé et al., 2021; González-Ravé et al., 2022).

However, evaluation of training periodization and altitude training with elite swimmers have not been reported in parallel, so it is difficult to identify the individual and combined effects of these training interventions. The aim of this collective case series study was to quantify parallel changes in performance, hematological and anthropometric effects of multiple altitude training camps in a cohort of elite swimmers.

## 2 Methods

### 2.1 Participants

The swimmers involved were four females (height 172 ± 6 cm; mass 62 ± 8 kg; age: 21 ± 2 yr; mean ± SD) and two male international swimmers (height 178 ± 11 cm; mass 74 ± 11 kg; age 22 ± 2 yr) trained by one coauthor of this study, and all were middle distance specialists. All swimmers were members of the Spanish National Team, and medalists in World and/or European Championships from 2011 to 2018 in short or long course competitions, classified as Tier 5 (World Class) or Tier 4 (International Level) according to the classification framework of McKay et al. (2022). The study was approved by the local ethics committee, and the swimmers and their coach provided written approval for retrospective analysis. A collective case series design was conducted in accordance with the Helsinki Declaration. A university ethics committee approved this research (UNNE-2020-010).

### 2.2 Experimental design

A collective case series is an observational descriptive study that follows a group of athletes who are undergoing the same or similar training over a certain period of time. As there is no experimental protocol or control training program for allocation of athletes (swimmers) to altitude training, coach and swimming support staff have to determine how these results translate to other groups of swimmers. Results of a collective case series may generate hypotheses and effect sizes that are useful in designing further studies, including randomized controlled trials. However, we acknowledge it is not appropriate to draw formal causal inferences from a case series on the effectiveness training interventions (Dekkers et al., 2012).

### 2.3 Procedure

A traditional periodization model was designed using three macrocycles covering the winter competition (December), national championships (April), and the main international competition (August) organized in 2016 (OG) and 2018 (European Championship). The aim of the first macrocycle was to develop general fitness and specific qualities oriented to the main event for each swimmer (100-200 m breaststroke, 200–400 m freestyle, 400 m individual medley). The goals of second and third macrocycles were to develop specific qualities required for the different events, culminating in the taper and competition. All the training intensity distribution performed in the pool was categorized into five intensity levels (Fernández-Lázaro et al., 2020). Intensities z1, z2, and z3 represented swimming speeds below (= 2 mmol l^–1^), equal to (= 4 mmol l^–1^), and slightly above (= 6 mmol l^–1^) the onset of blood lactate accumulation, respectively. High-intensity swimming that elicits blood lactate levels of ∼10 mmol l^–1^ was defined as intensity z4 and maximal intensity swimming as intensity z5 (González-Ravé et al., 2021).

**FIGURE 1:**
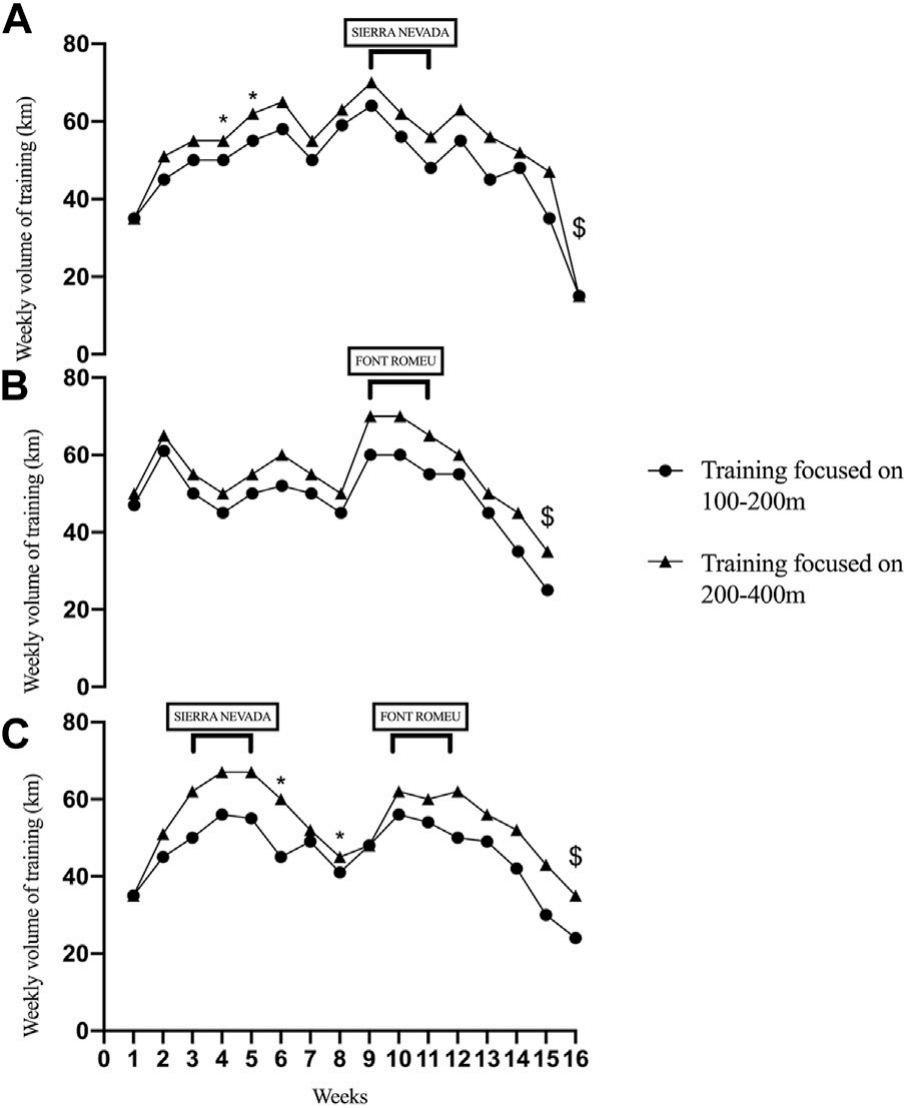
Weekly volume of training (km) in a season. Notes: **(A)** first macrocycle, **(B)** second macrocycle, **(C)** third macrocycle. *: competitions; $: main competition.

One of the female swimmers focused her training on 100–200 m, (see Figure 1), while the rest focused the training volume on 200–400 m. TID remained the same for all swimmers. According to the coach (one of the co-authors), gender does not affect any of these training parameters, such as volume and TID analyzed, except for dryland training, whose variables were not analyzed in this article.

The swimmers typically completed 3–4 scheduled altitude camps each season in the high-performance center in France (Font Romeu, 1850 m) and Spain (Sierra Nevada, 2,320 m). In a typical season with four altitude camps, the initial altitude camp in the first macrocycle was scheduled for 21 days in November. In the second macrocycle in February-March, another altitude camp was scheduled for 24 days. The third and final macrocycle involved two programmed altitude camps, one of them for 18 days in May, and the second for 21 days in June-July in 2016 and 2018. The volume of training was typically decreased in the two weeks of training prior to each altitude camp with spiking of higher intensity work in the Z3-Z4-Z5 zones (González-Ravé et al., 2021). In the altitude training camps, the volume increased compared to the previous two weeks, with an initial emphasis on volume at Z1-Z2 intensities before increasing work in the Z3-Z4-Z5 zones. Three graded incremental (7 × 200-m) swimming tests (one each macrocycle) was employed to quantify the training zones as a response to increasing speeds of swimming (Pyne et al., 2001).

The altitude training was interspersed with periods at home (sea level) to develop specific qualities required for the next altitude camp. This sequence was followed by a taper prior to competition. An example of volume and intensity distribution in Font Romeu is shown in Figures 1A–C. The timing of return from altitude prior to competition was between 20–32 days, with 28 days the most common duration.

Subjects were weighed to the nearest 0.1 kg using a Seca scale (Hamburg, Germany), and sum of six skinfold measurements used to indicate body fat (Dawson et al., 2002; Garrido-Chamorro et al., 2012). Anthropometric measurements were performed according to ISAK standards (Marfell-Jones et al., 2006). These measures were collected in the early morning before the first altitude camp in the second macrocycle, and the first day after the last altitude training camp in the third macrocycle.

A venous blood sample (4 mL) was drawn from an antecubital venipuncture early in the morning 3 days before the altitude training camp, and after the first day upon return to sea level. Blood samples were analyzed in duplicate for hemoglobin concentration (Radiometer OSM-3), hematocrit and red blood cells (spun capillary tubes) (Stray-Gundersen et al., 2001; Schena et al., 2002).

### 2.4 Statistical analysis

A descriptive analysis was performed using means, standard deviation, and percentage of change in performance, physiological or anthropometric measures. Changes in swimming performance were interpreted against the smallest worthwhile change (0.3%) in elite competition and with 95% confidence limits (CL) (Pyne et al., 2004).

## 3 Results

### 3.1 Performance outcomes

The female swimmers included a silver medallist in the 400 m freestyle, a finalist in the 200 m breaststroke at a FINA World Championships, and a third female a gold medallist in the 200 m backstroke at a European Championships. One of the male swimmers was a bronze medallist in the 400 m individual medal at a European Championships, and finalist in this event at an Olympic Games. The other male achieved his personal best in the national championship. The performance following the altitude training camps improved in terms of the personal best time 0.6% ± 0.8% (95% CL 0.1%–1.1%), season best time 1.6% ± 0.7% (95% CL 1.2% to 2.0%) and previous season best time 1.6% ± 0.5% (95% CL 1.3%–1.9%).

**FIGURE 2:**
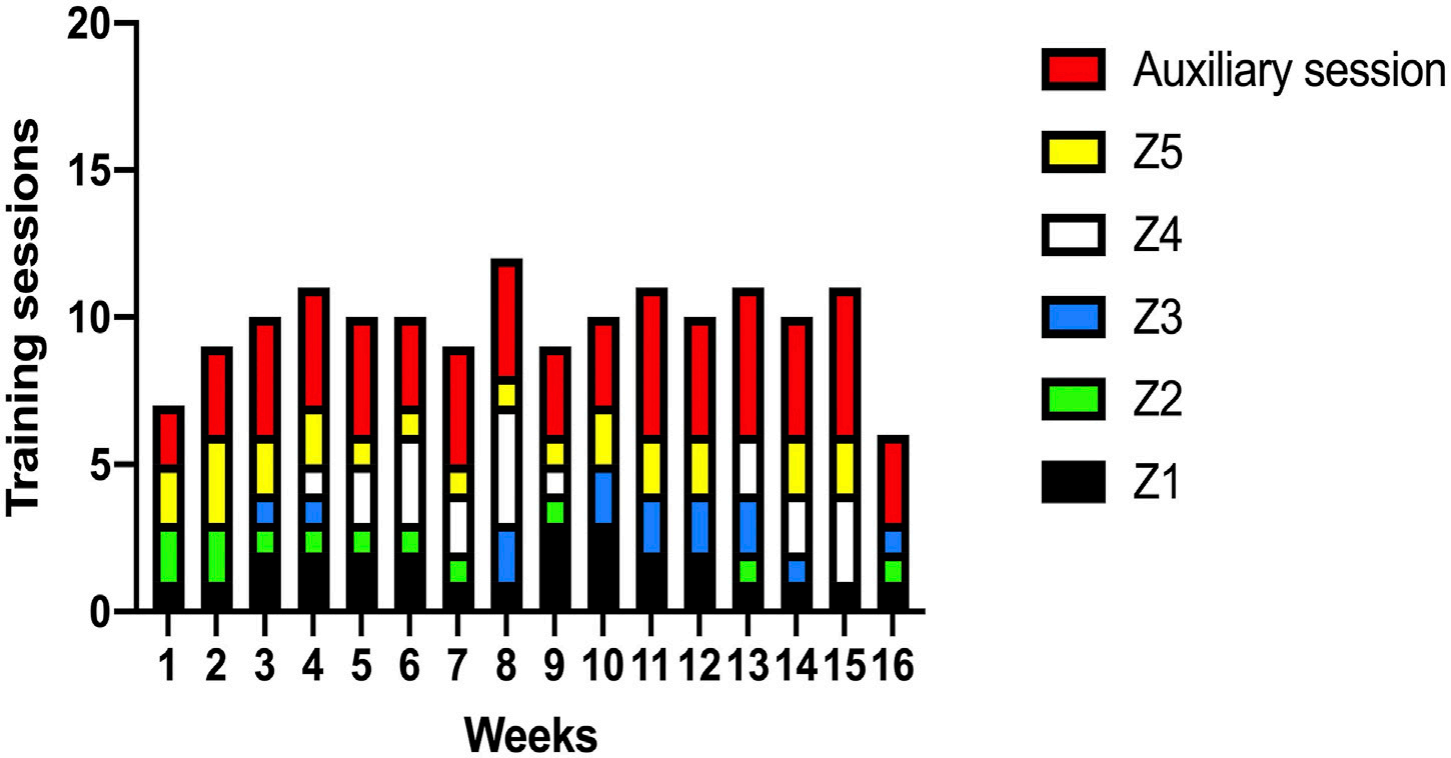
Training Intensity Distribution (TID) in 5 zones for each macrocycle (Main sessions). Notes: Z1: Zones 1 [ = 2 mmol 1-11]; Z2: Zones 2 [ = 4 mmol 1-1]; Z3: Zone 3 [ = 6 mmol 1-11]; ZA: Zone 4 [∼10 mmoll-1]; ZS: Zone 5 [maximum intensity swimming]. Auxiliary sessions includes TID in Z1 mainly, secondarily Z2, and technique.

Figures 1, 2 indicate the pattern of mean weekly volume and five zones of training intensity distribution (TID) for the swimmers analyzed in the whole season, divided into three macrocycles. The volume of macrocycle 1 follows a similar pattern of a traditional periodization, characterized by a progressive increase during the preparatory period, and then a modest reduction as training intensity increases. In the weeks 1-4 the early season was characterized by a build-up in training volume, followed by a cyclical pattern of increases and decreases in the mesocycles (typically 2–3 weeks in length), peaking at week 9 (7 weeks before competition), then a classic three-week taper into the competition. The total volume for macrocycle 1 was 768 km for training focused on 100–200 events and 862 km for training focused on middle-distance swimmers. The mid-season macrocycle followed a different distribution of the volume, with less variation in training volume in the first half of the cycle, but higher volumes in the Font Romeu altitude camp (weeks 10 and 11), before a sequential reduction with the taper. The total volume was 735 km for training focused on 100–200 events and 835 km for middle-distance swimmers.

Finally, the late-season macrocycle 3 leading to the international competition shows an initial four weeks similar to macrocycle 1. In weeks 6 and 8, a reduction in volume for regional competitions was made, and then three weeks at Font Romeu with lower training volumes in comparison with the previous macrocycle 2. The volume in this macrocycle was 729 km and 857 for sprinters and middle-distance swimmers respectively. A peak weekly volume of ∼70 km at altitude was completed by middle-distance swimmers, and ∼60 km for sprint swimmers. The TID shows a combination of both higher intensity quality sessions (Z4 and Z5), and auxiliary sessions (Z1 and Z2) for preparatory work, technique and recovery. Macrocycle 1 follows a polarized TID, with some early season Z5 high-intensity training in the first four weeks, then a focus on Z2-Z4 for a few weeks (weeks 5–8), then a focus on zone 4 training in the later weeks. Macrocycle two maintains a similar pattern with the TID. There is a more regular Zone 5 work through the season, then a late burst of Z4 in week 14. Macrocycle 3 also exhibits a polarized TID, with 9 sessions of Z5 in weeks 1-4, then a mesocycle of Z4 in weeks 5-8 and again weeks 13–15.

### 3.2 Anthropometry

The sum of six skinfolds reduced by 14.4% (95% CL 18.8%–9.9%) for the two males, and by 4.2% (95% CL 2.4%–9.2%) for two females. The mean difference between pre-post test measurements was −12.0 mm for males, and −8.1 mm for females preparing for the European Championships in 2018. Body mass decreased for all swimmers by 4.6% or 2.5 kg. Similar changes in anthropometric characteristics were observed for the FINA World Swimming Championships in 2013. The sum of six skinfolds for two female swimmers reduced by 15.8% (mean: -13 mm) (95% CL 19.5%–12.0%) and body mass decreased by 3.8% or 2.5 kg.

### 3.3 Blood analysis

Altitude training represented a substantial percentage of the season (23% of total weeks). The mean hemoglobin (Hb) value of the female swimmers during the seasons analyzed were 13.8 ± 1.1 g/100 mL. The mean Hb increased 5.8% (95% CL 2.4%–9.2%) from pre (13.3 ± 0.9 g/100 mL) to post (14.1 ± 0.7 g/100 mL) per season for the female swimmers. In the male swimmers, the mean hemoglobin (Hb) values were 15.6 ± 0.6 g/100 mL, with a modest decrease of 1.4% (95% CL -3.9%–1.2%) from pre (15.9 ± 0.5 g/100 mL) to post (15.7 ± 0.2 g/100 mL) season. The mean hematocrit values during the seasons analyzed were 41.6% ± 2.7% (females) and 46.1% ± 0.6% (males). The mean hematocrit increased 4.7% (95% CL -3.6%–2.7%) from pre (40.4% ± 2.2%) to post (42.3% ± 1.8%) for the females, and a decrease of 0.5% (95% CL -1.9%–1.1%) from pre (47.0% ± 1.8%) to post (46.8% ± 1.0%) for males. The mean red blood cells (RBC) values during the seasons analyzed were 4.5 ± 0.1 10^6^/mm^3^ (females) and 5.2 ± 0.1 10^6^/mm^3^ (males). The mean hematocrit increased 3.9% (95% CL -0.3%–8.0%) from pre (4.4 ± 0.1 10^6^/mm^3^) to post (4.6 ± 0.2 10^6^/mm^3^) for the females, with an increase of 1.9% (95% CL -1.9%–5.7%) from pre (5.2 ± 0.2 10^6^/mm^3^) to post (5.3 ± 0.2 10^6^/mm^3^) for males.

## 4 Discussion

The main outcomes of the present investigation were that a periodized altitude training program of three macrocycles elicited substantial improvements in the season-best time, personal best time, and selected haematological parameters. The elite swimmers who used three macrocycles with 3-4 altitude camps showed a good outcome in competitions after 47 weeks of training. Swimmers can benefit from the cumulative effects of multiple altitude camps over a single season. However, coaches should have in mind the importance of customizing these approaches by individualizing the programming of altitude training for different swimmers. It seems that a traditional periodized approach to altitude training, with cyclical increases in both training volume and intensity, can facilitate substantial improvements in physiological capacities and competition performance in international swimmers. Training loads were adjusted according to swimmers’ experience, age and distance specialists enhanced the ecological validity of this investigation.

The present results suggest the benefits of conducting integrated training camps at moderate altitudes with traditional periodization through the whole season. The performance in competitions improved in terms of the personal best time, season best time and previous season best time. Multiple altitude exposures in a season may be effective for enhancing the benefits of altitude training on competition performance in middle distance swimming events (Wachsmuth et al., 2013; Mujika et al., 2019). Altitude training camps are generally placed throughout the season before the main competition. Six to nine weeks prior to the main competition is a typical time to undertake altitude training (Wachsmuth et al., 2013; Mujika et al., 2019). Nevertheless, other training camps can be conducted in the middle of the macrocycles to emphasize both aerobic and anaerobic training contents. The timing of a peak performance following altitude training is likely to be influenced by a combination of altitude acclimatization and de-acclimatization responses. The periodization and responses to training and tapering conducted at and after altitude are key considerations for the coach (Hermosilla et al., 2021; González-Ravé et al., 2022).

Regarding the training volume per week, we reported higher volumes (km) per week than similar studies conducted in elite French swimmers. Hellard et al. (2019) showed that elite sprinters completed weekly training volume ranging between 29,000 and 37,000 m, and the middle-distance elite swimmers ranging 39,000–42,000 m. Similar volumes of training were reported by Pollock et al. (2019) in elite British swimmers (sprinters: 43,200 ± 530 m; middle distance swimmers: ∼50,000 m). However, the use of a polarized approach over the season yielded a better performance in World Class and international swimmers. An effective taper strategy with a decrease of overall training volume in the competitive period integrating multiple altitude exposures can elicit improvements in swimming performance (Mujika et al., 2002). The evidence from the present study showed that middle-distance swimmers accumulated larger volume during training than sprinters in the whole season. Polarized training may be a good option for sprinters and middle-distance elite swimmers when implemented appropriately (progressive load increases, sufficient macrocycle duration) in agreement with the study of Pla et al. (2019), who showed an improvement of 1% in 100 m performance for the polarized TID group of junior swimmers.

Prescription of training intensity is a key consideration for the coach and swimming scientist. The strategy of decreasing volume with more work at Z3 to Z5 two weeks before an altitude training camp, coupled with an increase of volume at Z1-Z2 appears a suitable strategy for promoting physiological and performance adaptations. Some authors recommend reducing the intensity (Daniels and Oldridge, 1970; Schmitt et al., 2018) and increasing the frequency of training sessions (Mujika et al., 2019) during the first week(s) of LHTH. The timing after return from altitude could have substantial effects on swim competition performance. Our results showed a worthwhile improvement of ∼1.6% of the season best time and the previous season time. We scheduled the return from altitude training camps between 20–32 days before a major swimming competition based on earlier recommendations (Wachsmuth et al., 2013; Mujika et al., 2019). Our results confirm research showing that multiple altitude exposures within a season interspersed by prolonged periods at sea level may increase in physiological effects gained at altitude (Saunders et al., 2009; Saunders et al., 2019).

There are mixed views of whether altitude training is an effective way for swimmers to optimize subsequent performance at sea level. Body mass and/or fat mass losses are not a consistent outcome in studies at the moderate altitudes with LHTH or LHTL training (2000–2,500 m) (Mujika et al., 2019). However, our results show a decrease in both fat percentage and body mass, most likely related to an increase of resting metabolic rate at moderate altitudes (Woods et al., 2017), and/or the additional training programmed at altitude. The data collected before the second macrocycle and after the third macrocycle are in line with the higher training volume through these macrocycles. Our results highlight that coaches should prioritize swimming performance more than other secondary variables such as anthropometry particular close to competition.

The approach of multiple exposures over the season allows repeating the stimulus for increases in Hb and non-hematological training adaptations. Repeated altitude exposure can decrease the initial negative effects of altitude ascents due to faster acclimatization *via* “hypoxic memory” (Song et al., 2017; Mujika et al., 2019). Our results confirm the approach that swimmers should tailor repeated altitude exposures to emphasize the training goals of the macrocycle (González-Ravé et al., 2021; Hermosilla et al., 2021). A period between 20–32 days seems to promote post-altitude performance peaking, but individualizing training will be important to identify the best time to compete after an altitude camp (Wachsmuth et al., 2013; Hermosilla et al., 2021). Pla et al. (2020) reported comparable improvements in performance with a similar intervention program in Font Romeu with elite swimmers. Despite improving swimming performance in the competitions, there were two males non-responders to hematological changes. The simplest explanation for these contrasting results may be the individual variability in hematological changes because four of the subjects showed an increase in RBC, Hb and mean hematocrit. Given the relationship between changes in haematological parameters and physiological determinants (e.g., V̇O_2max_) and performance, it is a prerequisite before and after altitude camp to monitor the physiological adaptations of swimmers. Being at altitude causes a reduction in blood O_2_ concentration, which triggers the overexpression of hypoxia-inducible factor 1 and regulates the body’s response to hypoxia *via* a systemic mechanism allowing these training adaptations by multiple physiological responses (Fernández-Lázaro et al., 2022).

Some limitations need to be acknowledged. Given that Tier 4 and 5 swimmers were involved in this study, it was not possible to recruit a control group with similar characteristics of participants. We also acknowledge the challenge of generalizing these case series study results to swimmer specializing in other swimming strokes and events of difference distances. The case series approach also has methodological limitations. The case series involve descriptive studies that do not test the hypothesis of training efficacy, neither is it a suitable design to draw formal causal inferences. Regarding altitude training and its effects on physical performance, physiological causality is partly based on erythropoiesis. Given the interrelationship between changes in Hb and endurance performance observed with altitude training, facilitating an increase in Hb during altitude exposure is often viewed as a required contributor towards a positive performance outcome following altitude training (Mujika et al., 2019). On this basis, it is more common to examine hematological effects of high altitude training on absolute hematological parameters like total hemoglobin mass (tHb) or blood volume that can show if marked erythropoiesis occurred. In our case we only had data on mean Hb, mean hematocrit and mean red cell concentration. Interpretation of hemoglobin and hematocrit measures with altitude training would be improved with parallel data on hydration and plasma volume changes because these data were retrieved from the coaches staff for monitoring training purposes. Swimmers can benefit from the cumulative effects of multiple altitude camps over a season, having in mind the individualization of programming of altitude training for different swimmers. Performance in swimming competitions improved in this case series in parallel with Hb and non-hematological training adaptations.

## 5 Conclusion

An integrated traditional periodization with three performance peaks over the season employing three to four altitude training camps around 21–24 days of duration, scheduling the last return between 20–32 days before the main competition, can improve swimming performance, hematological parameters, and body composition. Coaches should consider this research as a real-world scenario, reflecting the styles and philosophies of an experienced swimming coach of Olympic and World Championship swimmers.

## Data Availability

The raw data supporting the conclusions of this article will be made available by the authors, without undue reservation.
